# Association between nutritional status and dengue infection: a systematic review and meta-analysis

**DOI:** 10.1186/s12879-016-1498-y

**Published:** 2016-04-20

**Authors:** Nguyen Thi Huyen Trang, Nguyen Phuoc Long, Tran Thi Minh Hue, Le Phi Hung, Tran Dinh Trung, Doan Ngoc Dinh, Nguyen Thien Luan, Nguyen Tien Huy, Kenji Hirayama

**Affiliations:** Hue University of Medicine and Pharmacy, Hue City, Vietnam; Online Research Club, Institute of Tropical Medicine (NEKKEN), Nagasaki University, 1-12-4 Sakamoto, Nagasaki, 852-8523 Japan; University of Medicine and Pharmacy, Ho Chi Minh City, Vietnam; Hanoi Medical University, Ha Noi, Vietnam; Da Nang University of Medical Technology and Pharmacy, Da Nang city, Vietnam; Health Strategy and Policy Institute (HSPI), Ha Noi, Vietnam; Department of Clinical Product Development, Institute of Tropical Medicine (NEKKEN), Leading Graduate School Program, and Graduate School of Biomedical Sciences, Nagasaki University, 1-12-4 Sakamoto, Nagasaki, 852-8523 Japan; Department of Immunogenetics, Institute of Tropical Medicine (NEKKEN), Leading Graduate School Program, and Graduate School of Biomedical Sciences, Nagasaki University, 1-12-4 Sakamoto, Nagasaki, 852-8523 Japan

**Keywords:** Nutritional status, Dengue, Systematic review, Meta-analysis

## Abstract

**Background:**

Dengue infection has various clinical manifestations, often with unpredictable clinical evolutions and outcomes. Several factors including nutritional status have been studied to find the relationship with dengue severity. However, the nutritional status had conflicting effects on the complication of dengue in some previous studies. Therefore, we conducted a systematic review and performed a meta-analysis to analyze the association between nutritional status and the outcome of dengue infection.

**Methods:**

Eleven electronic databases and manual searching of reference lists were used to identify the relevant studies published before August 2013. At least two authors worked independently in every step to select eligible studies and extract data. Dengue severity in the included studies must be classified into three categories: dengue fever (DF), dengue hemorrhagic fever (DHF) and dengue shock syndrome (DSS).

**Results:**

Thirteen articles that met the inclusion criteria came to final analysis. A meta-analysis using fixed- or random-effects models was conducted to calculate pooled odds ratios (OR) with corresponding 95 % confidence intervals. It has shown that there was no statistically significant association between DHF group and DSS group in malnutritional and overweight/obesity patients with OR: 1.17 (95 % CI: 0.99–1.39), 1.31 (0.91–1.88), respectively. A significantly inverse relation between DF and DHF groups of malnutritional patients was revealed (OR = 0.71, 95 % CI: 0.56–0.90). Our meta-analysis also indicated a statistically significant negative correlation between malnourished children with dengue virus infection and healthy children (OR = 0.46, 95 % CI: 0.3–0.70). When analyzing patients with normal nutrition status, we found out that there was a significantly negative relationship between DHF and DSS groups (0.87; 95 % CI: 0.77–0.99). Other comparisons of DSS with DF/DHF groups, DSS/DHF with DF groups, and DHF with DF groups in normal nutritional patients showed no significant correlation. However, the findings should be interpreted cautiously because all significant associations were lost after removing of the largest study.

**Conclusions:**

Results from previous studies failed to show any solid consistency regarding the association between the nutritional status and dengue infection. Consequently, the effects of nutritional status on dengue disease outcome has been controversial. Further studies are recommended to clarify the impact of nutritional status on dengue infection.

**Electronic supplementary material:**

The online version of this article (doi:10.1186/s12879-016-1498-y) contains supplementary material, which is available to authorized users.

## Background

Dengue is an emerging disease in many parts of the tropics and subtropics of the world. The World Health Organization (WHO) approximates that about 2.5 billion people or 40 % of the world’s population live in dengue endemic countries. An estimated 50 to 100 million infections occur annually causing 22,000 deaths, most of which are children. The pattern of hyperendemic transmission of multiple dengue serotypes has now been established in Asia, the Pacific, the Americas, Africa, and the Caribbean [1]. Four distinct dengue viruses (dengue 1–4) have *Aedes aegypti* and *Aedes albopictus* as their principal vectors. All cause a similar clinical syndrome which ranges from primary dengue fever (DF) to severe dengue marked by hemoconcentration from vascular leakage in dengue hemorrhagic fever (DHF) and dengue shock syndrome (DSS) [2]. The development from non-severe to severe dengue could be unpredictable. Notwithstanding, early diagnosis and appropriate treatment may prevent further development and severity of the disease [2, 3].

It was documented that many previous studies examining the severity of dengue disease and nutritional status have resulted in controversial outcomes [4–13]. Some studies found that patients with excessive body weight were at increased risk for more severe DHF [6, 11] while malnutrition is a protective factor due to suppressed immune activation in malnourished children [10, 13]. In contrast, studies suggest that the nutritional status is unlikely to have an influence on the complication of dengue [4, 5, 7–9, 12]. Therefore, we conducted our systematic review and meta-analysis of relevant studies to determine the association of nutritional status with not only in DSS and DHF group [14] but also in the variety of dengue infection.

## Methods

Our study was conducted according to the recommendation of the PRISMA statement [15] (Additional file 1: Table S1. PRISMA checklist). The protocol for this review has been registered at PROSPERO International prospective register of systematic reviews (No. CRD42013005172).

### Literature searching and selection criteria

In August 2013, we searched for eligible studies from eleven electronic databases. PubMed and Information Sciences Institute (ISI) were searched by using the following query: dengue AND (nutrition OR nutritional OR malnutrition OR malnutritional OR “body mass index” OR obesity OR overweight). In Scopus, we used the search terms “TITLE-ABS-KEY(dengue AND (nutrition OR nutritional OR malnutrition OR malnutritional OR "body mass index" OR obesity OR overweight))”. In Google Scholar, studies were retrieved by this query: “allintitle: dengue (nutritional OR "malnutrition" OR "body mass index" OR "obesity" OR "overweight")”. We performed the search terms “dengue AND (nutritional OR "malnutrition" OR "body mass index" OR "obesity" OR "overweight")” on WHO Global Health Library and Popline. As for Indexing of Indian Medical Journals (IndMED), the following search terms were used: “dengue AND ((nutritional) OR (malnutrition) OR (BMI) OR (obesity) OR (overweight))”. In addition, we searched the keyword “dengue” on the remaining libraries including African Journals OnLine (AJOL), African Index Medicus (AIM), New York Academy of Medicine Grey Literature Report (NYAM) and System for Information on Grey Literature in Europe (SIGLE). Moreover, we manually collected studies by screening the references of relevant reviews and included studies [16–20].

All titles and abstracts (when available) were reviewed independently by at least two of seven authors (NTHT, NPL,TTMH, TDT, DND, LPH) after a pilot training of 20 % relevant articles with a senior researcher (NTH). Studies were considered eligible if they stated any information of dengue infected patients together with information of nutritional status. There were no restrictions with respect to publication language, patient age (children or adult) or study design. Non-English reports were translated into English by authors with the help of native international students at Nagasaki University. We excluded articles with the following characteristics: (i) including data that could not be reliably extracted; (ii) including data sets considered overlapping; (iii) letter, case report, review, thesis or conference paper; (iv) animal study or in vitro study without patients. Any conflicts were resolved by discussion and consensus between authors.

### Data extraction

Full-text versions of all eligible studies were obtained. Data were extracted by two independent reviewers (NTHT and NPL) and were checked by at least two of four authors (NTH, NTHT, NPL, TTMH). Any disagreement was resolved by discussion and consensus. The data extracted included the first author, year of publication, year of patient recruitment, study design (cross-section or case-control), data collection (prospective or retrospective), assignment of patients (consecutive or random), country and city of origin, hospital where the patients were recruited, characteristics of the patient population (infant, children or adult), number of included individuals, criteria of dengue infection (confirmed or clinical diagnosis), criteria of DSS, DHF and DF, data about nutritional status (malnutrition, normal or obesity) of included individuals. We also recorded a description of blinded interpretation of factors, the age of the patients and Body Mass Index (BMI).

Fifteen of thirty-three studies were included for extracting data at the first time. However, two studies did not have suitable data to perform meta-analysis [4, 21]. We contacted twice via email these authors to get more information but there was no reply. Consequently, thirteen studies were incorporated into final analysis.

### Quality assessment

Quality assessment was independently carried out by two investigators (NTHT, NPL). The quality of included studies was assessed based on the combined criteria suggested by Pai et al. [22] and Wells et al. [23]. The quality of each study was determined across eight metrics: study design, full description of characteristics of patient population (infant, children and adult), data collection (prospective or retrospective), assignment of the patients (consecutive or random), inclusion criteria, exclusion criteria, blinded interpretation of factors, and full description of dengue diagnosis. Quality was evaluated by discussion and consensus after the independent review of each study by four authors (NTH, NTHT, NPL, TTMH) (Additional file 2: Table S2).

### Meta-analyses

Meta-analyses including sensitivity, subgroup, meta-regression, and publication bias analyses was performed using Comprehensive Meta-analysis software version 2 (Biostat, USA, https://www.meta-analysis.com/) as previously described [14].

## Results

### Systematic review

**Fig. 1:**
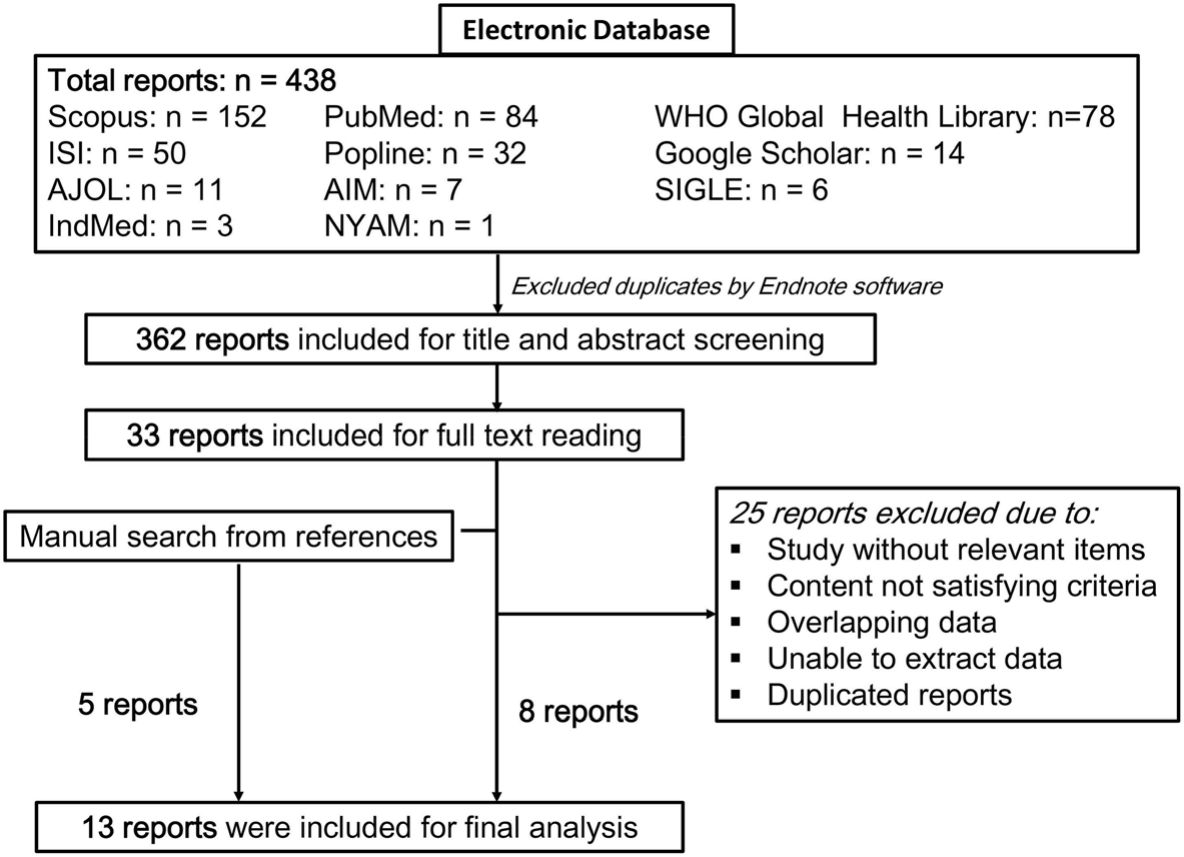
Flow diagram of the article screening process from electronic databases

After filtering and deleting the duplicates by Endnote software, a total of 362 publications were included for initial screening of titles and abstracts (Fig. 1). Full-text reading was continued in 33 studies that met the inclusion and exclusion criteria. We excluded 25 articles with the following reasons: (1) study without relevant items; (2) content not satisfying criteria; (3) overlapping data; (4) unable to extract data; (5) duplicated reports. Finally, 13 reports (8 studies from electronic databases and 5 studies retrieved from manual search) came into the final analysis.

Most of the included studies were performed in Asia (5 from Indonesia, 4 from Thailand, 2 from Viet Nam and 1 from India) while only one study was from El Salvador, a Latin American country [7, 8, 10, 12, 13, 16–20, 24–26]. The characteristics of these studies are outlined in Additional file 3: Table S3. We identified eight cross-sectional studies and five case-control studies. Ten studies were prospective and the remaining were retrospective or not mentioned (n = 3). The subjects of 13 selected researches were both children and/or infants. Serological diagnosis (ELISA or hemagglutination inhibition), PCR and viral isolation were some laboratory diagnosis methods used in dengue infection. All the thirteen studies used the WHO classification of dengue severity in which nine studies used the WHO 1997 criteria, two used WHO 1986, one used WHO 1999 and one study did not mention the particular year of WHO classification.

The definition of malnutritional status was different between studies. Nine studies used weight-for-age to assess malnutritional status [8, 10, 12, 13, 16, 18, 24–26]. Two of these nine studies [10, 25] used both weight-for-age and height-for-age while other three [7, 19, 20] did not give a clear method to evaluate the malnutrition status. In addition, three studies also used weight-for-height [10, 17] and BMI-for-age [25] to measure this factor.

### Malnutrition

**Fig. 2:**
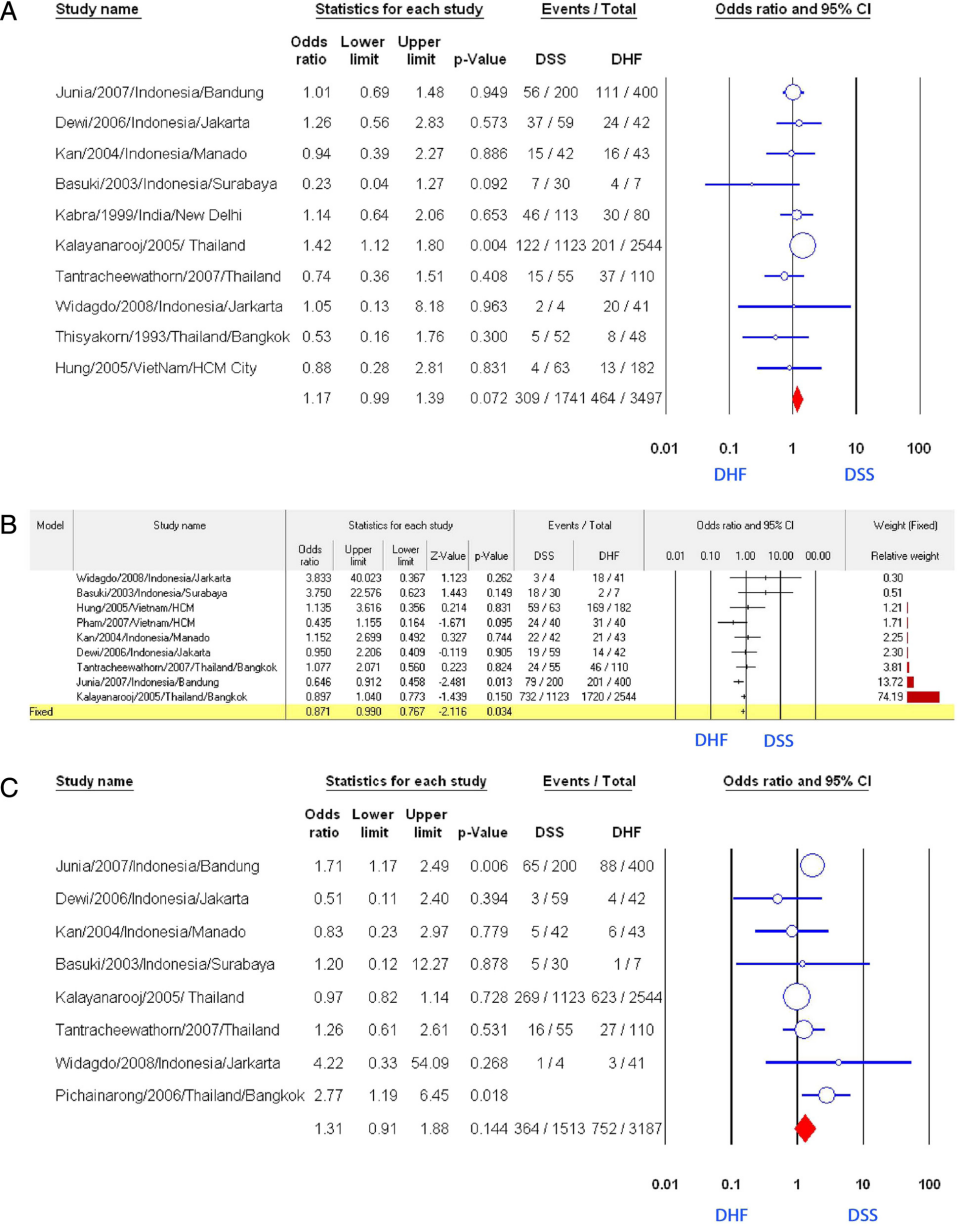
Association of nutritional factors and the severity of dengue. **a** Meta-analysis forest plot showing the pooled ORs of malnutrition between DSS and DHF group with 95 % CIs using fixed effect models. **b** Meta-analysis forest plot showing the pooled ORs of normal nutrition between DSS and DHF group with 95 % CIs using fixed effect models. **c** Meta-analysis forest plot showing the pooled ORs of obesity/overweight between DSS and DHF group with 95 % CIs using fixed effect models

**Fig. 3:**
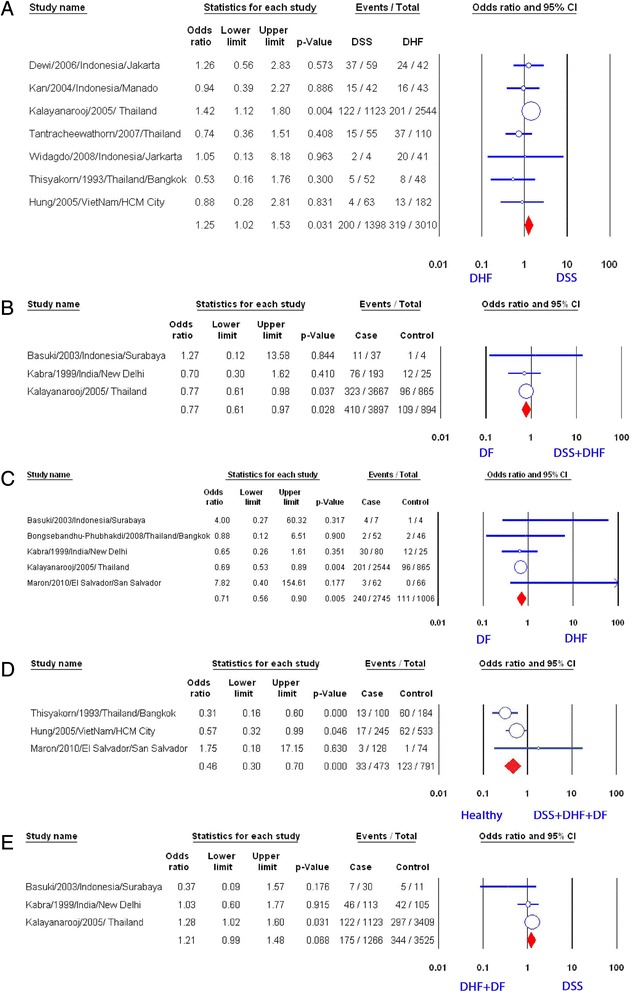
Association of malnutrition and the severity of dengue. **a** Meta-analysis forest plot showing the pooled ORs of malnutrition (weight for age) between DSS and DHF group with 95 % CIs using fixed effect models. **b** Meta-analysis forest plot showing the pooled ORs of malnutrition between DSS plus DHF group and DF group. **c** Meta-analysis forest plot showing the pooled ORs of malnutrition between DHF and DF group. **d** Meta-analysis forest plot showing the pooled ORs of malnutrition (weight for age) between any combined dengue and health control. **e** Meta-analysis forest plot showing the pooled ORs of malnutrition between DSS and DHF plus DF group

We analyzed 10 studies in which the authors made the comparison between DSS group and DHF group to evaluate the correlation between malnutrition and suffering DSS [7, 8, 10, 12, 13, 16–19, 26] (Fig. 2a). Pooling of these 10 studies revealed no significant correlation (OR = 1.17, 95 % CI: 0.99–1.39) (Table 1). Only the study of Kalayanarooj et al. showed a positive relation (OR = 1.42, 95 % CI: 1.12–1.80). Subgroup analysis of seven studies using nutrition assessment method by weight-for-age index showed that malnutrition status was related to DSS (OR = 1.25, 95 % CI: 1.02–1.53) [8, 10, 12, 13, 16, 18, 26] (Fig. 3a). However, removing the largest study of Kalayanarooj et al. [8] resulted in the loss of this correlation (*p* = 0.5). Three of the ten studies mentioned above permitted us to compare the ratio of malnourished children between DSS group and DHF plus DF group [7, 8, 19]. No significant correlation was established (OR = 1.21, 95 % CI: 0.99–1.48, Fig. 3e). When conducting a comparison between DSS plus DHF group to DF group by pooling these three studies, we detected an inverse correlation of malnutrition with developing hemorrhagic tendency (OR = 0.77, 95 % CI: 0.61–0.97). It is almost the same as the result of the study of Kalayanarooj et al. (OR = 0.77, 95 % CI: 0.61–0.98, Fig. 3b).

**Table 1:** Meta-analysis of the association between nutritional status and the severity of dengue. Pooled odds ratios (OR) with corresponding 95 % confidence intervals (95 % CI) of the published results were calculated where more than one study had investigated the factor

Variable	No. of study	Total sample size (DSS/DHF)	Heterogeneity		Model	Association with severity			Egger’s 2-tailed bias *p*-value
			*p*-value	*I* ^*2*^		*p*-value	Odds ratio (95 % CI)	*p*-value after removing 1 study	
Malnutrition (DSS vs. DHF) All methods	10	1741/3497	0 · 32	13	Fixed	0 · 072	1.17(0.99–1.39)		0.01
							1.27(1.09–1.49)^a^		
Malnutrition (DSS vs. DHF)	7	1398/3010	0 · 43	0	Fixed	0.031	1.25(1.02–1.53)		0.04
Weight for age							1.40(1.16–1.69)^a^		
Malnutrition (DSS + DHF vs. DF)	3	3897/894	0 · 89	0	Fixed	0.028	0.77(0.61–0.97)		
Malnutrition (DHFvs.DF)	5	2745/1006	0 · 38	4	Fixed	0.005	0.71(0.56–0.90)	0.84	0.12
Malnutrition (Combined dengue vs. healthy) (weight for age)	3	473/791	0.20	39	Fixed	<0.001	:0 · 46(0 · 30–0 · 70)		
Malnutrition (DSS + DHF vs. healthy) (weight for age)	2	345/717	0.17	47	Fixed	<0.001	:0 · 44(0 · 29–0 · 67)		
Normal nutrition (DSS vs. DHF) All methods	9	1616/3398	0 · 26	21	Fixed	0 · 03	0 · 87(0 · 77–0 · 99)	0 · 20	0 · 43
Obesity/overweight (DSS vs. DHF) All methods	8	1513/3187	0 · 05	51	Random	0 · 15	1 · 31(0 · 91–1 · 88)		0 · 32

^a^OR: adjusted odds ratio calculated after the addition of potential missing studies using the trim and fill method of Duvall and Tweedie

There were five studies evaluating DHF group and DF group to identify the relationship of malnutrition with developing DHF [7, 8, 19, 24, 25]. There was a significantly inverse relation (OR = 0.71, 95 % CI: 0.56–0.90). Among the five studies, only the study of Kalayanarooj et al. showed a significant relationship (Fig. 3c) and the pooled result became non-significant after removing the study of Kalayanarooj et al. [8] (data not shown).

Finally, meta-analyzing three studies [10, 13, 25] showed a statistically significant negative correlation of malnourished children with dengue virus infection with the healthy children (OR = 0.46, 95 % CI: 0.3–0.70, Fig. 3d, Additional file 4: Table S4). Both studies of Hung et al. and Thisyakorn et al. revealed negative correlations. However, these two studies compared DSS plus DHF group with healthy group [10, 13], while other compared DHF plus DF group with healthy group [25].

### Normal nutrition

Pooling nine studies [8, 10, 12, 16–20, 26], all of which were performed in South-East Asia, indicated that children with normal nutrition were inversely associated with DSS compared to DHF (OR: 0.87, 95 % CI: 0.77–0.99, Fig. 2b). Neither evidence of heterogeneity (*p* = 0.26, *I*^*2*^ = 21) nor publication bias (*p* = 0.43) existed. None of the nine studies revealed a significantly positive association of normal nutrition with DSS while one study showed a clearly negative relation of this factor with DSS [17]. Moreover, further analysis of eight studies after excluding any of three studies [8, 17, 20] did not provide a significant association of normal nutrition with DSS.

**Fig. 4:**
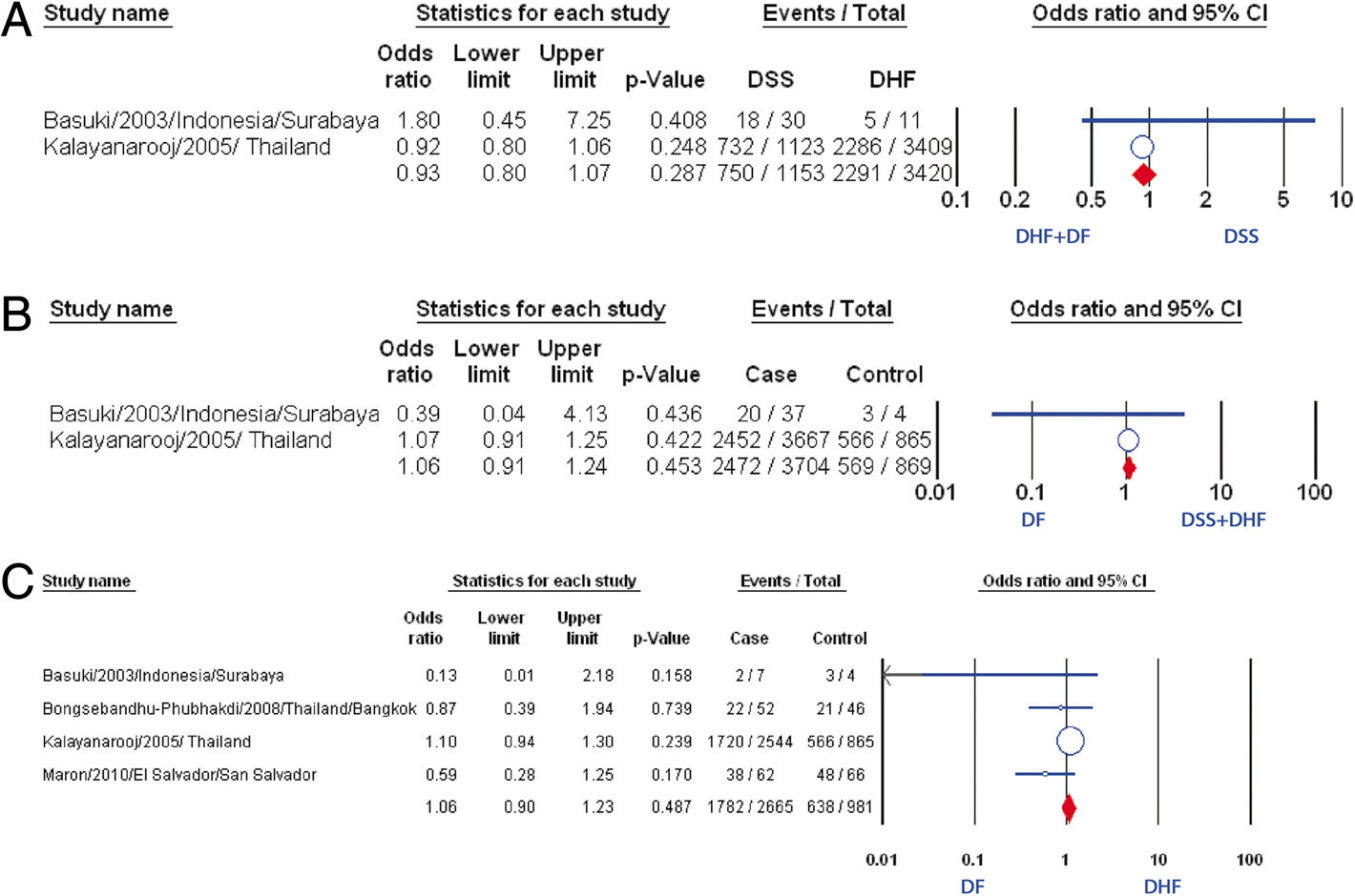
Association of normal nutrition and the severity of dengue. **a** Meta-analysis forest plot showing the pooled ORs of normal nutrition between DSS and DHF plus DF group. **b** Meta-analysis forest plot showing the pooled ORs of normal nutrition between DSS plus DHF group and DF group. **c** Meta-analysis forest plot showing the pooled ORs of normal nutrition between DHF group and DF group

When comparing group of DSS with group of DF/DHF (Fig. 4a), no correlation between normal nutrition and DSS was found (OR: 0.93, 95 % CI: 0.8–1.07) after pooling from two studies [8, 19]. Similarly, when comparing group of DSS/DHF with group of DF, the analysis did not find a statistically significant relation of normal nutrition with developing hemorrhagic tendency or shock (OR: 1.06, 95 % CI: 0.91–1.24, Fig. 4b). Moreover, a meta-analysis of four studies [8, 19, 24, 25] showed no significant association with normal nutrition when comparing DHF with DF (OR: 1.06, 95%CI: 0.90–1.23, Fig. 4c).

### Overweight and obesity

**Fig. 5:**
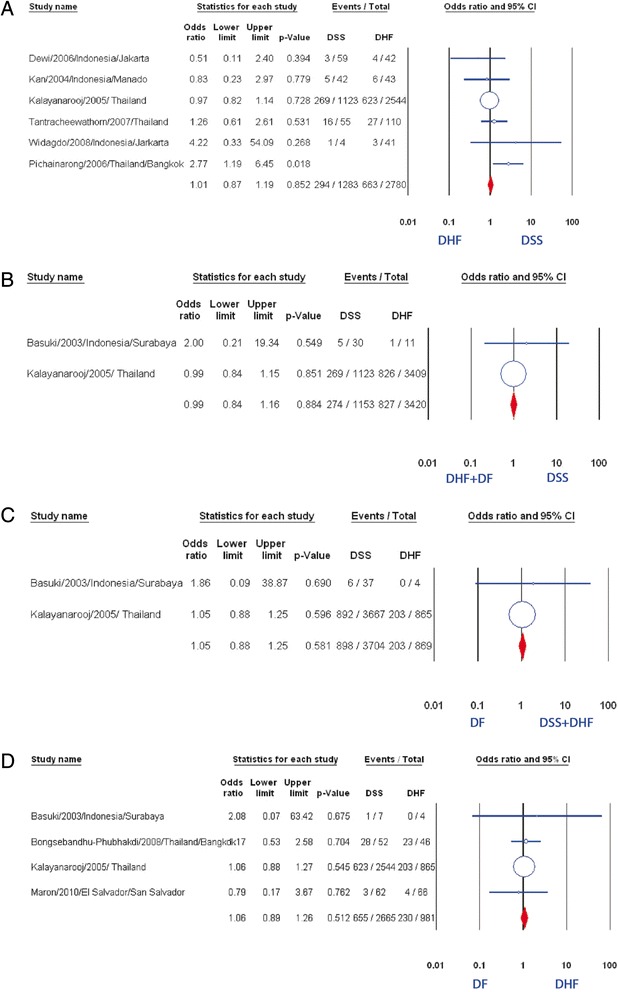
Association of obesity/overweight and the severity of dengue. **a** Meta-analysis forest plot showing the pooled ORs of obesity/overweight (weight for age) between DSS group and DHF group. **b** Meta-analysis forest plot showing the pooled ORs of obesity/overweight between DSS and DHF plus DF group. **c** Meta-analysis forest plot showing the pooled ORs of obesity/overweight between DSS plus DHF group and DF group. **d** Meta-analysis forest plot showing the pooled ORs of obesity/overweight between DHF group and DF group

Pooled result from eight studies [8, 11, 12, 16–19, 26] revealed no significant association of overweight/obesity with DSS compared with DHF (OR: 1.31, 95 % CI: 0.91–1.88, Fig. 2c), although two studies showed positive correlation [11, 17]. There was no evidence of publication bias (p = 0.32). Further subgroup analysis of six studies which used weight-for-age index did not find any statistically significant correlation between two factors mentioned above (OR: 1.01, 95 % CI: 0.87–1.19, Fig. 5a) [8, 11, 12, 16, 18, 26].

When we compared group of DSS with group of both DHF and DF, no correlation of overweight/obesity with suffering DSS was found (OR: 0.99, 95 % CI: 0.84–1.16, Fig. 5b). We got the same result when comparing group including DSS and DHF with group of DF (OR: 1.05, 95 % CI: 0.88–1.25, Fig. 5c). Besides the study of Basuki et al. and the study of Kalayanarooj et al., there are two additional studies in which the authors compared between DF and DHF patients [24, 25]. The pooled result showed that overweight/obesity was not found associated with the dengue severity (OR: 1.06, 95 % CI: 0.89–1.26, Fig. 5d).

## Discussion

It was thought that malnutrition is a protective factor against DSS, due to immune dysfunction [27–29]. Nevertheless, our pooled result of all relevant studies suggested no relation between malnutrition and DSS. Further subgroup analysis of the studies that assessed malnutrition by weight-for-age index, in contrast, showed a positive correlation (Fig. 3a). This correlation may be explained by the small volume of extracellular fluid and intravascular fluid in the malnourished patients, making them more likely to suffer DSS when plasma leakage occurred [30]. Other factors including host genetic factor, dengue virus serotype and genotypes [31–39], and using different methods to evaluate nutritional status may play a role and may explain why our pooled results were not homologous to the result of subgroup analysis. However, excluding the study of Kalayanarooj et al., which had a very large study population [8], led to a loss of association of malnutrition with DSS. More well-designed prospective studies using anthropometric indices are required to confirm this correlation.

Nutrition is now generally considered as an important determinant for immune responses, while malnutrition is considered to impair the host defense [14]. However, our result showed that malnutritional status may be a protective factor against development of DF/DHF (Fig. 3c), mostly due to the effect of a very large study by Kalayanarooj et al. [8]. Further studies in the future may be needed to clarify the exact protective mechanism of malnutrition against dengue infection.

Many scientists believed that normal nutrition is a risk factor of DSS [27–29]. However, our meta-analysis found that normal nutritional status may be a protective factor against DSS, and no original study in our included analysis indicates a significant positive correlation (Fig. 2d). Euvolemia and larger extracellular volume in children with normal nutrition may be the reason that explains this protective effect [30]. Once again, this negative association should be interpreted with caution, because there are three studies strongly affecting the overall result, and removing any of these studies led to the loss of association [8, 17, 20].

In the pool of relevant studies, no correlation between overweight/obesity and DSS was established. Similarly, we did not find any association of overweight/obesity with DHF, unlike Halstead’s hypothesis in which obese children are “expected to have a stronger immune response and are higher risk of developing DHF than normal children” [8]. Nowadays, obesity is considered chronic, low-grade inflammation, with excess production of IL-1β, Il-6 and TNF-α [40, 41]. According to Milner and Beck, “It is possible that chronic exposure to pro-inflammatory cytokines may desensitize immune cells to inflammatory responses during an actual infection” [42]. However, the exact effect of obesity/overweight on the immune system during dengue infection is unknown. Further studies are needed to resolve this question.

## Conclusions

In summary, there are still many debates about the effect of nutritional status on dengue infection. More studies may be carried on to identify the association of nutritional status with dengue virus infection.

### Availability of data and materials

All data is presented within the manuscript and its supplementary files.

### Ethics approval and consent to participate

Not applicable.

### Consent for publication

Not applicable.

## Additional files

Additional file 1: Table S1. PRISMA checklist. (DOCX 23 kb) Additional file 2: Table S2. Scoring system for quality assessment of selected studies. (DOCX 19 kb) Additional file 3: Table S3. Characteristic of studies included in this meta-analysis. (DOCX 39 kb) Additional file 4: Table S4. Full meta-analyses of severity of dengue with nutritional status that were investigated in at least two studies. Pooled odds ratios (OR) with corresponding 95 % confidence intervals (95 % CI) of the published results were calculated where more than one study had investigated the factor. (DOCX 25 kb)

## Abbreviations

95 % CI: 95 % confidence intervals
AIM: African index medicus
AJOL: African journals on line
BMI: body mass index
DF: dengue fever
DHF: dengue hemorrhagic fever
DSS: dengue shock syndrome
NYAM: New York Academy of Medicine Grey Literature Report
OR: odds ratio
PRISMA: the preferred reporting items for systematic reviews and meta-analyses
SIGLE: System for Information on Grey Literature in Europe
