# Supplementary material for: Association between nutritional status and dengue infection: a systematic review and meta-analysis
Source: BMC Infect Dis. 2016 Apr 20;16:172. doi: 10.1186/s12879-016-1498-y (PMC4839161; doi:10.1186/s12879-016-1498-y)
Supplement: Additional file 2: Table S2. — Scoring system for quality assessment of selected studies. (DOCX 19 kb) [file 12879_2016_1498_MOESM2_ESM.docx]

**Additional file 2: Table S2.** **Scoring system for quality assessment of selected studies**

| Criteria | 0 point | 1 point |
| --- | --- | --- |
| Study design | case or no description | all case |
| Characteristic of patient population (infant, children, adult) | no description | full description |
| Data collection | retro or no description | prospective |
| Assignment of the patient | not consecutive, not random, no description | consecutive or random |
| Inclusion criteria | no description | full description |
| Exclusion criteria | no description | full description |
| Interpretation of factors | not blinded or no description | description of blinded method |
| Dengue diagnosis |  | full description |
