# Supplementary material for: Association between nutritional status and dengue infection: a systematic review and meta-analysis
Source: BMC Infect Dis. 2016 Apr 20;16:172. doi: 10.1186/s12879-016-1498-y (PMC4839161; doi:10.1186/s12879-016-1498-y)
Supplement: Additional file 3: Table S3. — Characteristic of studies included in this meta-analysis. (DOCX 39 kb) [file 12879_2016_1498_MOESM3_ESM.docx]

**Additional file 3: Table S3. Characteristic of studies included in this meta-analysis.**

| Author/Publication year/Country/City | Data year | Study design ^†^ | DSS | DHF | DF | Health control | Subject of study | Data collection^‡^ | Recruitment^¶^ | Diagnosis of dengue^#^ | Criteria of dengue ^**^ | Quality score |
| --- | --- | --- | --- | --- | --- | --- | --- | --- | --- | --- | --- | --- |
|  |  |  | (n) | (n) |  |  | I: Infant |  |  |  |  |  |
|  |  |  |  |  |  |  | C: Children |  |  |  |  |  |
|  |  |  |  |  |  |  | A; Adult |  |  |  |  |  |
|  |  |  |  |  |  |  | Range, mean± |  |  |  |  |  |
| Junia/2007/Indonesia/Bandung [[17](#_ENREF_17)] | 2004-2005 | case | 200 | 400 | 0 | 0 | C (7.1y±3.2; 7.3y±3.5) | ND | ND | Non-confirmed included | WHO | 4 |
| Dewi/2006/Indonesia/Jakarta[[16](#_ENREF_16)] | 2003-2004 | cross | 59 | 42 | 0 | 0 | I,C (5m-15y) | retro | conse | Confirmed ELISA | WHO 1997 | 5 |
| Kan/2004/Indonesia/Manado[[18](#_ENREF_18)] | 2000 | cross | 42 | 43 | 0 | 0 | C (2y-12y) | prosp | conse | Confirmed ELISA | WHO 1997 | 7 |
| Basuki/2003/Indonesia/Surabaya[[19](#_ENREF_19)] | 2000-2001 | cross | 30 | 7 | 3 | 0 | C (2y-13y) | prosp | conse | Non-confirmed and confirmed cases (ELISA) | WHO 1997 | 6 |
| Bongsebandhu-Phubhakdi/2008/Thailand/Bangkok[[24](#_ENREF_24)] | 2004-2006 | cross | 0 | 52 | 46 | 0 | C (1y-15y) | prosp | conse | PCR, D-dimer, immune response to confirm | WHO 1997 | 7 |
| Kabra/1999/India/New Delhi[[7](#_ENREF_7)] | 1996 | cross | 113 | 80 | 25 | 0 | I, C (4m - 13y) | prosp | conse | Confirmed ELISA, IIFA, virus | WHO 1986 | 6 |
| Kalayanarooj/2005/ Thailand/Bangkok[[8](#_ENREF_8)] | 1995-1999 | case^a^ | 1123 | 2544 | 865 | 734 | C (7.9± 3.8y) | retro | conse | Confirmed ELISA & HI | WHO 1997 | 4 |
| Tantracheewathorn/2007/Thailand[[12](#_ENREF_12)] | 2003-2005 | cross | 55 | 110 | 0 | 0 | I, C (6m-14.9y) | prosp | random | immune response | WHO 1997 | 6 |
| Widagdo/2008/Indonesia/Jarkarta[[26](#_ENREF_26)] | 2005 | cross | 4 | 41 | 0 | 0 | I,C (75±35m) | prosp | conse | ND | WHO 1999 | 6 |
| Thisyakorn/1993/Thailand/Bangkok[[13](#_ENREF_13)] | 1993 | case^a^ | 52 | 48 | 0 | 184 | I,C (3m-15y) | prosp | conse | HI and/or virus isolation | WHO 1986 | 5 |
| Hung/2005/VietNam/Hochiminhcity[[10](#_ENREF_10)] | 1997-2002 | case^a^ | 63 | 182 | 0 | 533 | I, 6.8m (1m - 11m) | prosp | conse | Confirmed ELISA, HI | WHO 1997 | 5 |
| Marón/2010/El Salvador/San Salvador[[25](#_ENREF_25)] | 2004 | case^a^ | 0 | 62 | 66 | 74 | C (5y-12y) | prosp | conse | Confirmed ELISA | WHO 1997 | 6 |
| TBPham/2007/Vietnam/Hochiminhcity[[20](#_ENREF_20)] | 2005 | case^a^ | 40 | 40 | 0 | 0 | C(2y-14y) | prosp | conse | Confirmed ELISA | WHO 1997 | 6 |

ND: Not Description

^†^_:_ Cross: cross-section; case: case-control

^‡^: Retro: retrospective; prosp: prospective

^¶^: Conse: consecutive

^#^: IIFA: indirect immunofluorescence assay; HI: Haemagglutination inhibition test

^**^WHO 1997 criteria[[2](#_ENREF_2)]:

DF: Acute fever with two or more of Fever with two or more signs of headache, retro-orbital pain, mayalgia, arthralgia, rash, hemorrhages, and leukopenia accompanied with confirmed dengue virus infection tests.

DHF Grade I: DF signs plus a positive tourniquet test, thrombocytopenia (platelet count< 100,000 cells/mm^3^), and evidence of plasma leakage

DHF Grade II: Above clinical symptoms and laboratory signs plus spontaneous bleeding

DSS Grade III: Above clinical symptoms and laboratory signs plus circulatory failure signs of rapid, weak pulse with low pulse pressure (<20mmHg) or hypotension

DSS Grade IV: Profound shock with undetectable blood pressure and pulse

We defined Grade I and II as DHF, Grade III and IV as DSS.

WHO 1999 criteria [[31](#_ENREF_31)]:

DF: An acute biphasic fever with headache, myalgias, arthralgias, rashes and leucopenia and confirmed by virus isolation and/or serology.

DHF grade I: Fever accompanied by non-specific constitutional symptoms; the only haemorrhagic manifestation is a positive tourniquet test.

DHF grade II: Spontaneous bleeding in addition to the manifestations of Grade I patients, usually in the form of skin and/or other haemorrhages.

DHF grade III: Circulatory failure manifested by rapid and weak pulse, narrowing of pulse pressure (20 mmHg) or hypotension

DHF grade IV: Undetectable blood pressure and pulse.

We assigned Grade I and II as DHF, while Grade III and IV as DSS.
