# Supplementary material for: Association between nutritional status and dengue infection: a systematic review and meta-analysis
Source: BMC Infect Dis. 2016 Apr 20;16:172. doi: 10.1186/s12879-016-1498-y (PMC4839161; doi:10.1186/s12879-016-1498-y)
Supplement: Additional file 4: Table S4. — Full meta-analyses of severity of dengue with nutritional status that were investigated in at least two studies. Pooled odds ratios (OR) with corresponding 95 % confidence intervals (95 % CI) of the published results were calculated where more than one study had investigated the factor. (DOCX 25 kb) [file 12879_2016_1498_MOESM4_ESM.docx]

**Additional file 4: Table S4. Full meta-analyses of severity of dengue with nutritional status that were investigated in at least two studies.** Pooled odds ratios (OR) with corresponding 95% confidence intervals (95%CI) of the published results were calculated where more than one study had investigated the factor.

| Variable | No. of study | Total sample size  (DSS/DHF) | Heterogeneity | | Model | Association with severity | | | Egger’s 2-tailed bias p-value | Included studies (References list shown in Table S2) |
| --- | --- | --- | --- | --- | --- | --- | --- | --- | --- | --- |
|  |  |  | p-value | *I^2^* |  | p-value | Effect size (95% CI) | p-value after removing 1 study |  |  |
| Malnutrition (DSS vs. DHF)  All methods | 10 | 1741/3497 | 0·32 | 13 | Fixed | 0·072 | 1.17(0.99-1.39)  1.27(1.09-1.49) |  | 0.01 | [10,40,61,70,73,79,82,172,194]  Add Nutritional status of children with Dengue heamorrhagic Fever |
| Malnutrition (DSS vs. DHF)  Weight for age | 7 | 1398/3010 | 0·43 | 0 | Fixed | 0.031 | 1.25(1.02-1.53)  1.40(1.16-1.69) |  | 0.04 |  |
| Malnutrition (DSS vs. DHF+DF) | 3 | 1398/3010 | 0·21 | 37 | Fixed | 0.07 | 1.21(0.99-1.48) |  |  |  |
| Malnutrition (DSS+DHFvs.DF) | 3 | 3897/894 | 0·89 | 0 | Fixed | 0.028 | 0.77(0.61-0.97) |  |  |  |
| Malnutrition (DHFvs.DF) | 5 | 2745/1006 | 0·38 | 4 | Fixed | 0.005 | 0.71(0.56-0.90) | 0.84 | 0.12 |  |
| Malnutrition (Combined dengue vs.healthy) (weight for age) | 3 | 473/791 | 0.20 | 39 | Fixed | <0.001 | :0·46(0·30-0·70) |  |  |  |
| Malnutrition (DSS+DHFvs.healthy) (weight for age) | 2 | 345/717 | 0.17 | 47 | Fixed | <0.001 | :0·44(0·29-0·67) |  |  |  |
| Normal nutrition  (DSS vs. DHF)  All methods | 9 | 1616/3398 | 0·26 | 21 | Fixed | 0·03 | 0·87(0·77-0·99) | 0·20 | 0·43 | [10,40,61,70,79,82,132,172,194] |
| Normal nutrition  (DSS vs. DHF)  weight for age | 6 | 1616/3398 | 0·83 | 0 | Fixed | 0·24 | 0·92(0·80-1·06)  0·90(0·79-1·04) |  | 0·026 |  |
| Normal nutrition  (DSS vs. DHF+DF) | 2 | 1153/3420 | 0·35 | 0 | Fixed | 0·29 | 0·93(0·80-1·07) |  |  |  |
| Normal nutrition  (DSS+DHF vs.DF) | 2 | 3704/869 | 0·41 | 0 | Fixed | 0·29 | 1.06(0·91-1·24) |  |  |  |
| Normal nutrition  (DHF vs.DF) | 4 | 3704/869 | 0·18 | 39 | Fixed | 0·49 | 1.06(0·91-1·24) |  |  |  |
| Obesity/overweigh  (DSS vs. DHF)  All methods | 8 | 1513/3187 | 0·05 | 51 | Random | 0·15 | 1·31(0·91-1·88) |  | 0·32 | [10,40,70,79,82,133,172,194] |
| Obesity/overweigh^#^  weight for age | 6 | 1283/2780 | 0·15 | 38 | Fixed | 0·15 | 1·02(0·87-1·19) |  | 0·38 |  |
| Obesity/overweigh  (DSS vs. DHF+DF) | 2 | 1153/3420 | 0.55 | 0 | Fixed | 0·88 | 0.98(0·84-1·16) |  |  |  |
| Obesity/overweigh  (DSS+DHF vs.DF) | 2 | 3704/869 | 0.71 | 0 | Fixed | 0·59 | 1.05(0·88-1·25) |  |  |  |
| Obesity/overweigh  (DHF vs.DF) | 4 | 2665/881 | 0.95 | 0 | Fixed | 0·51 | 1.06(0·89-1·26) |  |  |  |

^a^OR: adjusted odds ratio calculated after the addition of potential missing studies using the trim and fill method of Duvall and Tweedie.
